# Case report: The Montgomery T tube may be the preferred transition option for achieving a smooth extubation after tracheotomy when complicating airway pathology is present

**DOI:** 10.3389/fmed.2025.1457903

**Published:** 2025-01-24

**Authors:** Jieqiong Wang, Xun Li, Weihua Xu, Nenghui Jiang, Bo Yang, Ming Chen

**Affiliations:** ^1^Department of Pulmonary and Critical Care Medicine, Tongde Hospital of Zhejiang Province, Hangzhou, Zhejiang, China; ^2^College of Medicine, Jiaxing University, Jiaxing, Zhejiang, China; ^3^Department of Anesthesia Operating Room, The Second Affiliated Hospital of Jiaxing University, Jiaxing, Zhejiang, China; ^4^Department of Pharmacy, Tongde Hospital of Zhejiang Province, Hangzhou, Zhejiang, China

**Keywords:** tracheotomy, Montgomery T tube, tracheostomy cannula, suprastomal granulomas, case report

## Abstract

Prolonged retention of tracheostomy tubes post-procedure often leads to complications, including granulation tissue overgrowth, airway narrowing, and laryngeal edema, necessitating delayed removal of the tracheostomy tube. Currently, a definitive therapeutic regimen capable of simultaneously resolving these complications and expediting tracheostomy decannulation remains elusive. Herein, we present an efficacious strategy addressing these airway morbidities and facilitating rapid tube removal. A 44-year-old male patient, who had undergone tracheostomy due to underlying disease, demonstrated substantial recovery following rehabilitation and was poised for tracheostomy tube extraction. However, bronchoscopic examination revealed severe granulation tissue at the stoma site and laryngeal edema, posing challenges to immediate decannulation. To tackle these issues concurrently while aiming for swift tube removal, we performed bronchoscopic intervention for granulation tissue excision, subsequently replacing the conventional tracheostomy tube with a Montgomery T tube as a transitional measure to restore normal ventilation. With additional rehabilitation fostering respiratory function enhancement, follow-up bronchoscopies confirmed no recurrence of granulations and significant reduction in laryngeal edema, thereby enabling the successful removal of the Montgomery T tube 2 months later, restoring the patient’s unassisted respiratory capacity. This case underscores a clinically pertinent insight: following resolution of local airway abnormalities impeding tracheostomy decannulation, the strategic implementation of a Montgomery T tube as a transitional phase merits serious consideration among clinicians managing patients with long-term tracheostomies. Our findings contribute to the development of more streamlined approaches to overcoming complexities associated with tracheostomy tube removal in clinical practice.

## Introduction

For patients undergoing tracheotomy requiring an indwelling tracheostomy tube, the ultimate goal is early and safe extubation. In addition to conditions which limit extubation, complications associated with a long-term indwelling tracheostomy cannulae can occur such as infection, granulation tissue hyperplasia (1), subglottic stenosis, vocal cord paralysis, vocal cord edema, airway softening, and trachea-esophageal fistula can also lead to delayed extubation or extubation and reintubation (2, 3). Airway pathology complications, especially when Suprastomal granuloma is combined with severe vocal fold edema, further increases management difficulty. The need to address both the complications and to achieve the goal of early tracheostomy tube extubation undoubtedly complicates subsequent treatment strategy.

Here, we report the case of a patient with significant granulation tissue hyperplasia combined with severe glottic edema following tracheotomy with an indwelling tracheotomy tube. To facilitate early extubation, we performed a bronchoscopic excision of the granuloma followed by placement of a Montgomery T tube to replace the original tracheostomy tube as a transition before allowing the patient to resume normal respiration. After 2 months of rehabilitation, bronchoscopic examination showed no new edema and edema in the vocal cord area was significantly reduced. At that time the Montgomery T tube was removed and normal respiration was successfully restored. A search of the clinical literature reveals that similar cases involving this type of procedure have not been reported to date.

## Case presentation

A 41-year-old male patient was admitted to the emergency ICU on April 10, 2022, with a diagnosis of “brainstem hemorrhage” resulting in “impaired consciousness for 1 day.” He had a history of long term uncontrolled hypertension which resulting from failure to regularly take his medication. Due to his critical condition upon admission, tracheal intubation was performed and mannitol was given to reduce intracranial pressure along with several other treatments. Because long term ventilation was anticipated, a tracheotomy was performed on April 20, 2022 to maintain adequate respiratory support. After the initial treatment, the patient regained consciousness, blood oxygenation increased and the muscle strength in the left limb was determined to be grade 1 at that time. Ventilation was discontinued on April 26, 2022 and replaced with high-flow oxygen through the tracheotomy cannula. The patient was transferred to the Department of Respiratory and Critical Care Medicine on May 23, 2022. During this period, the patient experienced “hospital-related pneumonia,” “urinary tract infection” and “catheter associated infection,” however all of these conditions ameliorated following appropriate treatment. During this time, the patient became more lucid and was able to perform simple directed activities. At this point, the patient was recovery progress suggested that removal of the tracheostomy tube would permit normal respiration. However, in order to more accurately assess the condition of the airway, promptly manage possible airway complications, and reduce the risks associated with the removal of the tracheostomy tube, we performed the following procedure on July 1, 2022 under general anesthesia.

**Figure 1:**
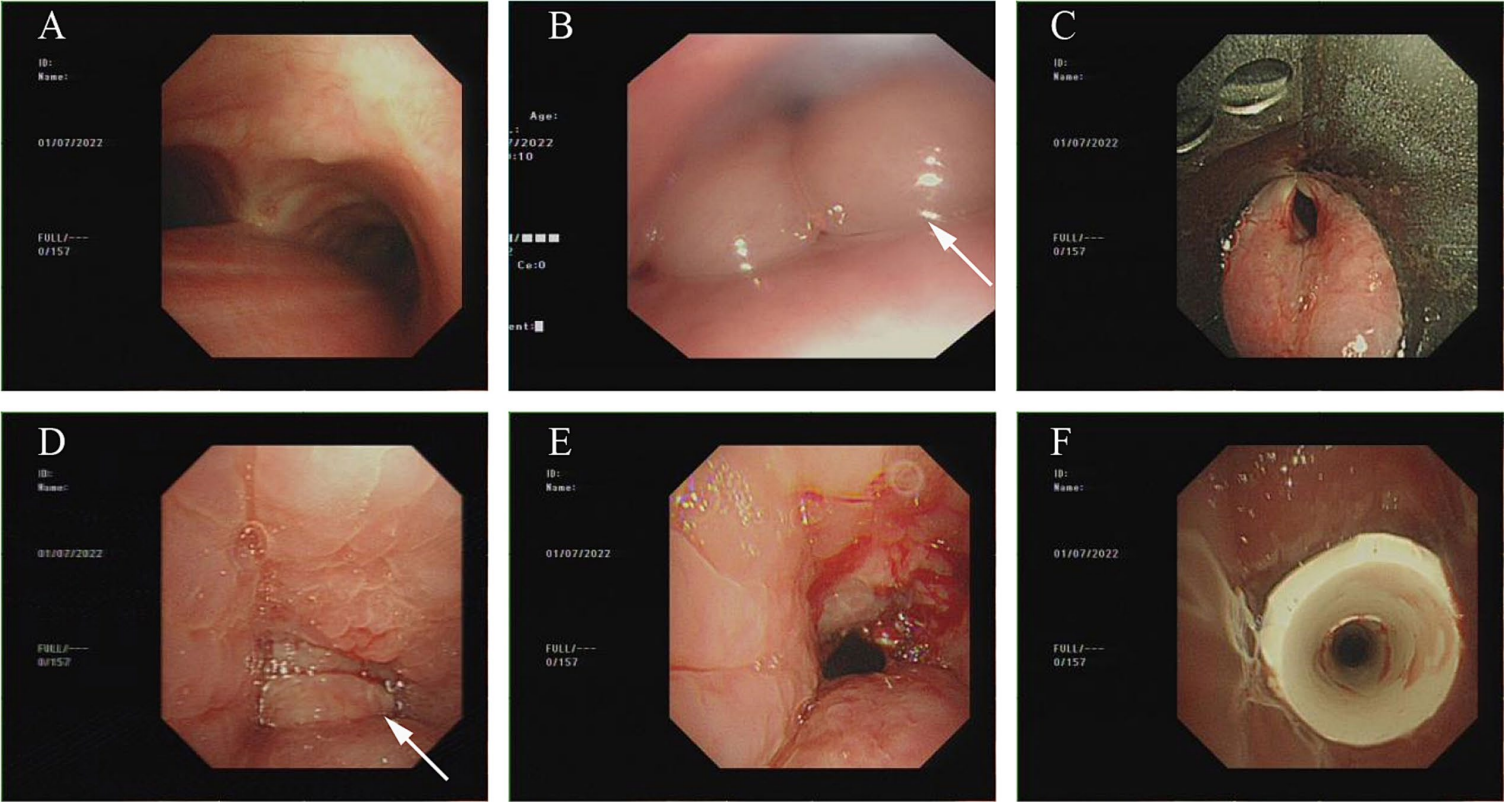
Bronchoscopy condition as of July 1, 2022. **(A)** No obvious abnormality were noted in the main airway below the tracheostomy sleeve. **(B)** Severe edema noted in throat and glottic region. **(C)** A view of severe glottal edema with support laryngoscope. **(D)** A Suprastomall granuloma obstructs the airway. **(E)** The majority of the granulomas were removed using the bronchoscope snare device allowing partial opening of the upper airway. **(F)** Montgomery T tube was inserted.

First, a ventilator was connected to the tracheostomy cannula used for respiratory support. A fiberoptic bronchoscope was introduced into the passage way of the tracheostomy tubing. This examination revealed no observable abnormalities at the lower end of the tube and in the distal extent of airway (Figure 1A).

Next, the bronchoscope was inserted through the nose to explore the pharyngeal airway region above the tracheostomy tube. It was found that severe edema and local tissue disfiguration in the pre-tracheal pharyngeal region (Figure 1B) did not permit, after several attempts, positive identification of the tracheal opening.

During the procedure, we requested that otolaryngologist use the laryngoscope observe the tissue of swollen glottic area (Figure 1C), expose the upper airway of the Tracheotomy incision. More granulomas were found in the upper airway which blocked almost the entire airway (Figure 1D).

Given the complications associated with the airway granulomatous lesions and glottic edema we hoped to remove the tracheostomy tube as soon as possible so that the patient could resume normal respiration. Therefore, an emergency multidisciplinary consultation was held with the anesthesiologist and otolaryngologist.

After consulting with them, we decided to use the laryngoscope to maintain a working channel within the airway. The granulomas were first removed using a fiberoptic bronchoscopic snare device (Figure 1E).

After removing the majority of the granuloma and establishing airway patency, the original PVC tracheostomy tube was removed and an 11-gauge Montgomery tracheal T tube was inserted (Figure 1F).

The horizontal branch of Montgomery T tube was left open postoperatively due to remaining edema in the vocal fold region. The horizontal branch of the Montgomery T tube was then plugged after edema in glottal area had subsided. The patient was evaluated to determine whether he could tolerate upper airway ventilation and was encouraged to talk and eat to simulate normal physiological activity. Around 2 weeks postoperatively, the patient was able to breathe when the Montgomery T tube horizontal branch was plugged. However, there were still excessive secretions in the patient’s airway at that time, so the horizontal branch was opened regularly for fluid aspiration.

**Figure 2:**
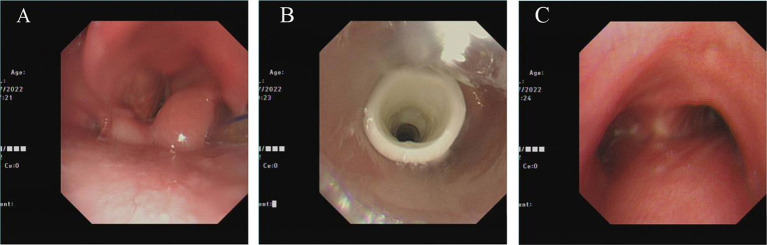
Bronchoscopy condition as of July 13, 2022. **(A)** Glottic edema was reduced, but was still obvious. **(B)** Montgomery T tube in optimal position. **(C)** No new granuloma appeared in the main part of the airway below Montgomery T tube.

We performed a routine bronchoscopy on July 13, 2022. Microscopically we saw a partially diminished edematous condition of the glottis and surrounding tissues (Figure 2A), a well-positioned Montgomery T tube (Figure 2B), and no new granulomas in the airway (Figure 2C).

**Figure 3:**
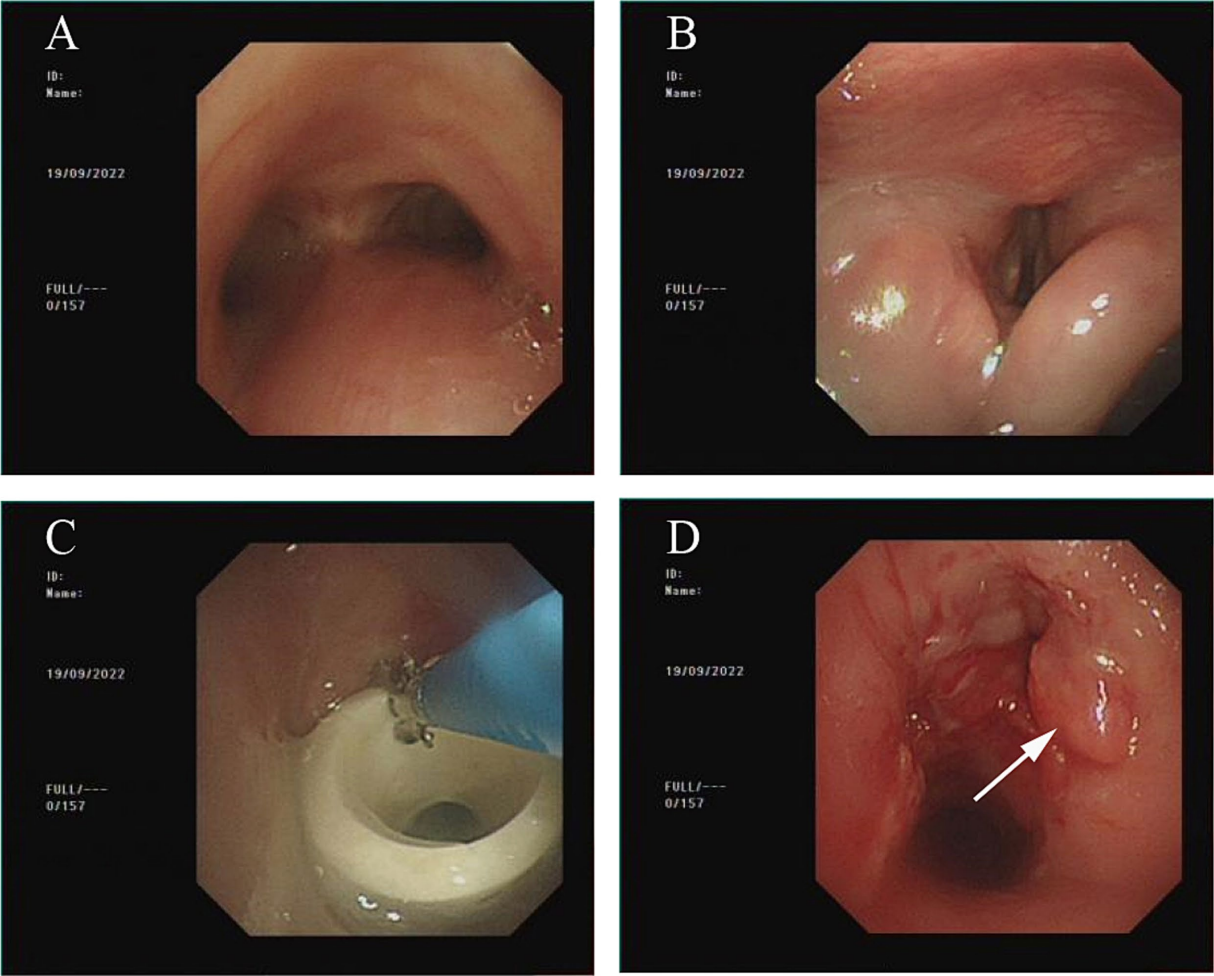
Bronchoscopy condition as of September 19, 2022. **(A)** No new granulomas noted in the airway below Montgomery T tube. **(B)** Glottic edema has notably subsided, but is not completely normal at this point. **(C)** Remove Montgomery T tube using biopsy forceps. **(D)** After removal of Montgomery T tube, the airway was essentially smooth.

At this time, some edema remained in the glottic area (Figures 3A,B), but it did not appear to markedly affect the patient’s breathing, so the Montgomery T tube was removed (Figures 3C,D). After this procedure, the patient recovered well and was able to breathe normally. At this point, the patient showed no dyspnea. The patient and their family expressed satisfaction with this.

## Discussion

There is a lack of consensus regarding when a tracheostomy tube can be safely removed. Some studies suggest that extubation should be considered once the patient no longer requires mechanical ventilation, airway obstruction has been resolved, airway secretions are controlled, and swallowing function is largely restored (4, 5). Utilizing a standardized extubation protocol to guide the removal of tracheostomy tubes can reduce extubation time, decrease the failure rate, and minimize complications, thereby providing greater benefits to patients (6). Clearly, early and safe extubation is beneficial to the patient.

It has been shown that endoscopy to assess the presence of airway stenosis prior to the removal of the tracheostomy tube can help improve the success rate of extubation (7). Patients undergoing tracheotomy have a high probability of developing airway abnormalities. Common complications include granulation tissue hyperplasia and subglottic stenosis (8). Some researchers believe that most granulomas can be detected by bronchoscopy and that they do not significantly impact airway patency, thus not requiring special treatment. However, for patients with granulomas causing significant obstruction who require immediate extubation, aggressive management of these granulomas is necessary. In such cases, direct endoscopic resection is generally the method of choice (8, 9).

While the patient management protocol described above focuses on the attendant complications, the ultimate goal of our treatment was not only to relieve the airway obstruction caused by granulomas, but to take steps to remove the tracheostomy tube as soon as possible when sarcoidosis was anticipated to accompany the presence of an indwelling tracheotomy tube. Long-term placement of tracheostomy tubes has been shown to promote the formation of granulomas within the airway. This phenomenon is associated with the physical irritation caused by the presence of the tube and the excessive, non-physiological mechanical stress exerted on the airway walls (10).

When granulation tissue hyperplasia is combined with severe edema in the vocal area, subsequent treatment becomes further complicated. Even after the granulomas are eliminated, the tracheostomy tube should not be removed immediately. This is because severe asphyxiation can occur if the artificial lumen closes before the normal breathing passage reopens due to severe supraglottic edema (11).

When considering the risk of granuloma recurrence with a PVC tracheostomy cannula, a metal tracheostomy cannula can be used during the transition period. Metal tubes are usually made of silver or stainless steel and offer several advantages: durability, inhibition of bacterial growth, non-reactivity with surrounding tissue, resistance to biofilm formation, and ease of cleaning (12). A plug test can be performed to determine whether the metal tracheostomy cannula can be safely removed. However, some investigators disagree on whether the plugging test is a reliable indicator for removal of the tracheostomy cannula (13, 14).When edema is present in the vocal fold region, it is not possible to accurately determine whether effective respiration can be maintained after removing a metal tracheostomy tube. Additionally, the inability to speak affects the patient’s quality of life. Rapid implementation of speech capabilities allows for more effective communication, improves swallowing function, and promotes recovery (14, 15).

Glucocorticoids have been shown to be an effective treatment during the acute phase of vocal fold edema (16, 17). However, there is no clear evidence that corticosteroids are beneficial for treating chronic vocal fold edema (18). This is because the edema only subsides after an extended period, and long-term hormonal therapy may increase the risk of complications such as secondary infections. Premature removal of the tracheostomy tube may necessitate re-intubation. Conversely, unnecessarily delaying extubation can lead to longer hospital stays and increased medical costs.

Therefore, the purpose of this report in describing the treatment of airway maintenance complications is not to focus on specific treatment measures but to present options for selecting appropriate treatment strategies. These strategies should not only effectively address urgent airway maintenance issues but also create optimal conditions for rapid extubation and the restoration of normal airway patency.

The insertion of the Montgomery T tube is primarily used to treat subglottic tracheal stenosis. It serves a dual role in supporting airway maintenance and stabilizing tracheal scarring (19). The Montgomery T tube is generally employed to address subglottic stenosis resulting from endotracheal intubation and tracheotomy. It is also utilized for airway stenosis caused by rare conditions such as polychondritis (20) or glycogen storage disease (21). Additionally, the Montgomery T tube can be used both for short-term treatments and long-term management of chronic conditions like airway malacia (22).

At present, no studies have evaluated the efficacy of using a Montgomery T tube as a transitional device prior to the removal of a standard PVC tracheostomy tube or the specific circumstances that warrant its use before extubation. To provide a comprehensive comparison, we have outlined the advantages and disadvantages of PVC tracheostomy tubes versus Montgomery T tubes in Table 1.

**Table 1:** Comparison of advantages and disadvantages of PVC tracheostomy tubes and Montgomery T tubes.

Characteristics/Parameters	PVC tracheostomy tube	Montgomery T tube
Replacement	Frequent, easy	Rarely needed, but complex if required
Airway complications	Common: mucosal injury, scarring, granulation, bleeding	Fewer complications; risk of stent fracture or displacement
Prevention of aspiration	Effective	Higher risk of aspiration pneumonia
Mechanical ventilation	Can connect to ventilator	Cannot connect to ventilator
Airway humidification	Requires enhanced humidification	Lower humidification needs
Microbial colonization	Higher risk	Lower risk
Speech and quality of life	Requires speaking valve, less esthetic	Easier speech, more esthetic

We believe that for patients experiencing airway maintenance complications such as suprastomal granuloma combined with glottic edema, bronchoscopic granuloma resection followed by the placement of a Montgomery T tube can be beneficial prior to extubation. The Montgomery T tube’s elasticity, toughness, and strong support help maintain structural integrity during long-term contact with the airway. Additionally, it exhibits good tissue compatibility and is non-toxic. Its optimal diameter, which is smaller than the airway diameter, effectively reduces irritation to the airway mucosa (23), thereby significantly decreasing the recurrence of granuloma formation at the tracheotomy incision and reducing glottic edema (24). It also allows for relatively flexible ventilation options. Approximately 2–3 days postoperatively, the horizontal branch can be closed to simulate normal respiration, depending on the resolution of inflammation, to assess whether severe edema in the vocal area impacts normal breathing. In this case, patients can attempt to breathe normally through the vertical branch about 1 week after the operation. Even with visible glottic edema, blood oxygen saturation can be maintained at over 90% at rest, suggesting that cannula removal may be possible even when some edema is still present. In our case, there was still some glottic edema when the Montgomery T tube was finally removed, but it did not affect normal respiration.

Additionally, the unique structure of the Montgomery T tube allows for the clearance of airway secretions through the lateral hole when the horizontal branch is open. When the horizontal branch is closed, patients can still speak through the vertical branch. This feature improves the patient’s quality of life and facilitates smoother extubation.

In summary, this report describes the treatment of airway complications using specially selected indwelling tracheostomy cannulas. The treatment strategy outlined here can provide valuable guidance for physicians conducting respiratory interventions in similar cases.

## Data Availability

The raw data supporting the conclusions of this article will be made available by the authors, without undue reservation.
